# Dataset on commuting patterns and mode-switching behavior under prospective policy scenarios for public transport

**DOI:** 10.1016/j.dib.2019.104703

**Published:** 2019-11-01

**Authors:** Cristian Toşa, Tomio Miwa, Takayuki Morikawa

**Affiliations:** a School of Civil Engineering, Technical University of Cluj-Napoca, Romania; b Institute of Materials and Systems for Sustainability, Nagoya University, Japan; c Institute of Innovation for Future Society, Nagoya University, Japan

**Keywords:** Travel behavior, Experimental design, RP/SP mode choice model, Transport policy

## Abstract

This paper covers a broadly used methodology used in travel behavior research aiming at determining individual and alternative-specific variables that influence the choice of the transportation mode for commuting trips. Data used in the analysis were obtained in July 2015 by means of a computer-assisted telephonic interview survey conducted in Cluj Metropolitan Area, Romania. The survey collected a wide range of day-by-day travel patterns, socioeconomic data, and attitudes and perceptions toward urban transportation services. Given the lack of studies from emerging, post-socialist countries, the survey assigned a section dedicated to an alternative ticketing policy for public transport services in order to evaluate the willingness of commuters to switch to a more sustainable transportation through non-coercive interventions. A revealed preference – stated preference modelling methodology was adopted in order to reveal the role of socioeconomic characteristics, along with features of transport supply and built environment in explaining commuting patterns and forecast sustainable modal splits. Both the survey and the methodology are scalable and flexible to be used, adapted, and applied in a wide range of transport policies regarding modal shifting strategies.

**Table:** Specifications Table

Subject	Social Sciences
Specific subject area	Transportation, Travel Behavior Analysis
Type of data	Table
How data were acquired	Computer-Assisted Telephonic Interview (CATI) survey
Data format	Raw Analyzed Filtered
Parameters for data collection	The survey addressed individuals older than 18, employee active status, living or residing in Cluj Metropolitan Area, and willing to share their current travel behavior and response to public transport improvement policy.
Description of data collection	The respondents were accessed by phone following Random Digit Dialing procedure in order to ensure demographic and spatial coverage for the sample. The respondents were asked to participate in a 15-min telephonic interview on matters of travel behavior.
Data source location	City/Town/Region: Cluj Metropolitan Area, Transylvania Country: Romania
Data accessibility	Data accompanies the current article.
Related research article	Cristian Toşa, Hitomi Sato, Takayuki Morikawa, Tomio Miwa Commuting behavior in emerging urban areas: Findings of a revealed-preferences and stated-intentions survey in Cluj-Napoca, Romania Journal of Transport Geography DOI 10.1016/j.jtrangeo.2018.02.011

**Table:** 

**Value of the Data** • Data contains commuter level information on current and prospective travel behavior, socioeconomic characteristics, derived spatial features, and attitudinal data for transport services in emerging urban areas. • Stakeholders with interest in transport policies can be provided with important insights on public response to non-coercive actions with regards to sustainable transportation in urban areas. • The empirical study could serve as a reliable framework for testing transportation policies, using the same or different modal attributes of interest. • The data brings additional value in explaining travel behavior in emerging urban areas in the context of post-communist countries from Central and Eastern Europe.

## 1 Data

**Fig. 1:**
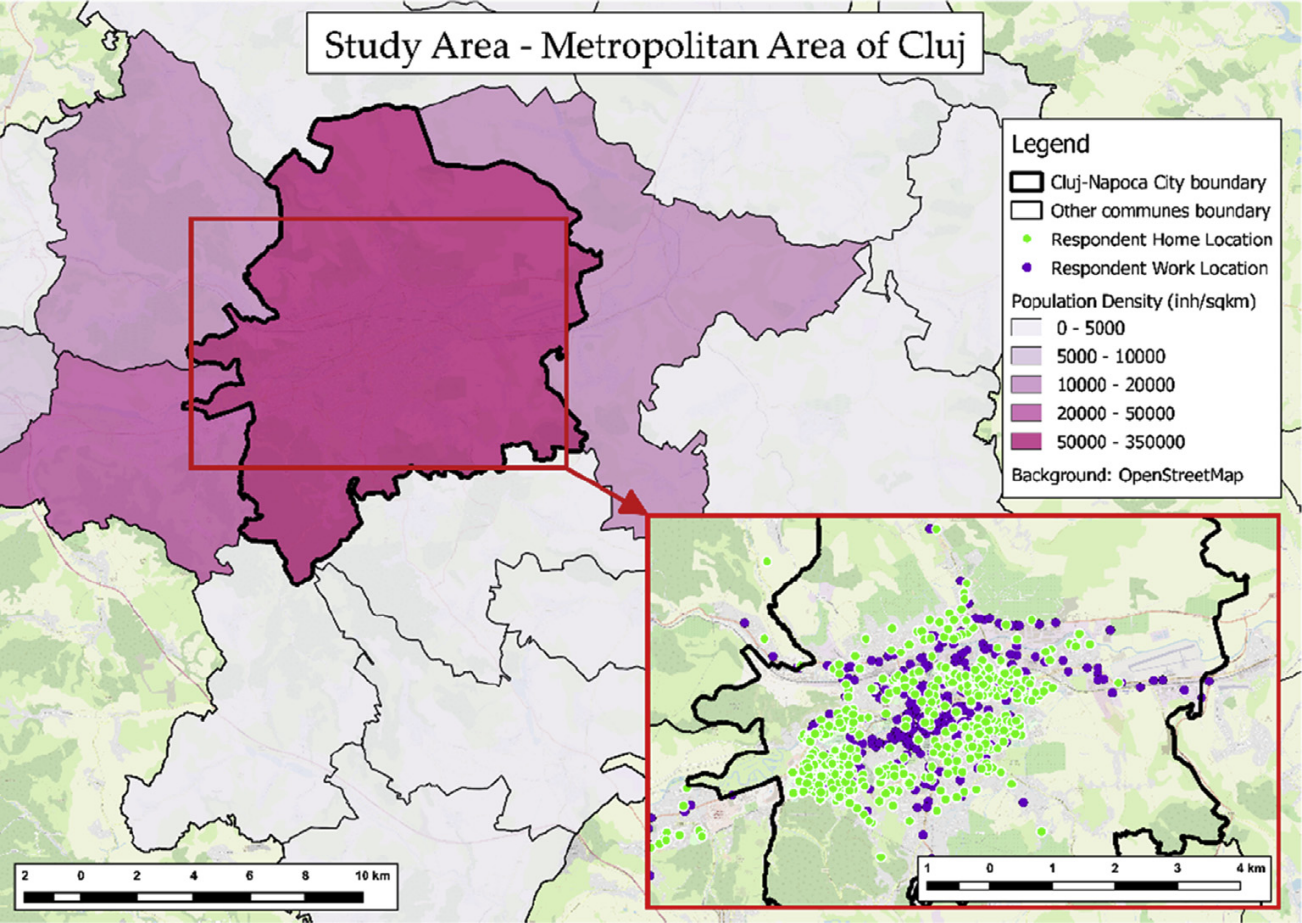
Study area.

The dataset described in this paper contains socio-economic and demographic characteristics, travel behavior, associated spatial features, and attitudinal indicators. Data was collected using a stratified sample representing the daily, morning rush-hour commuters from the Metropolitan Area of Cluj, Romania (Fig. 1). Several studies were previously conducted [1,2] with regards to data collection from individuals for the assessment of sustainable urban mobility in post-socialist urban areas [3], but data was scarce, and studies were exploratory. Toșa et al. [4] conducted a comprehensive study by revealing the generational differences and their demographic, socioeconomic, and attitudinal characteristics in quantitatively explaining commuting patterns within Cluj Metropolitan Area, Romania. To those were added elements of transport supply and built environment that contribute to the refinement of the choice process. The associated data and the methodology will be described in the following. The cleaned and processed dataset is part from a total of 1079 respondents who participated in the data collection process, and it comprises of a quota of 544 individuals (50.42%).

The socio-economic and demographic section in the questionnaire collected information related to the respondent, such as on gender, family size, age, education level and occupation, marital status, driver's license, household type, and income and. Table 1 integrates these characteristics by their associated levels and shows the percentage distribution. For each variable, the sum of the elements sample sizes equals the final quota of the respondents, i.e. 544 individuals.

**Table 1:** Socio-economic and demographic characteristics.

Variable	Element	Sample	Percentage
Gender	Male	283	52.02
	Female	261	47.98
Family size	Alone	37	6.8
	2	151	27.76
	3	180	33.09
	4 or more	176	32.35
Age cohorts	18–25	33	6.07
	26–35	138	25.37
	36–45	161	29.6
	46–55	142	26.1
	>55	70	12.87
Education	Bachelor and below	197	36.21
	Higher education	347	63.79
Occupation	Public worker	169	31.07
	Private worker	375	68.93
Marital status	Married	144	26.47
	Not married	400	73.53
Driver's license	Yes	426	78.31
	No	118	21.69
Household type	Individual home	123	22.61
	Apartment	421	77.39
Average income household (in RON)	<1000	31	5.7
	1001–2000	168	30.88
	2001–3000	133	24.45
	3001–4000	77	14.15
	4001–5000	53	9.74
	5001–6000	26	4.78
	>6000	56	10.29
Monthly transportation expenditure (in RON)	<50	22	4.04
	51–100	118	21.69
	101–200	155	28.49
	201–300	93	17.1
	301–400	57	10.48
	>400	99	18.2

The commuting behavior section of the questionnaire addressed questions to track the transport modes used for commuting and their corresponding weekly frequency. The transport modes included both motorized modes, such as public transport, car, motorcycle or taxi, and non-motorized modes, such as bicycle, and walking. These travel modes were selected for the questionnaire in order to emphasize their impact on traffic congestion and level of service. Therefore, modes such as motorcycle, car, and taxi represent personal motorized modes, and walking and bicycle represent non-motorized modes. The frequency data was processed in order to obtain the representative transport mode used for commuting, i.e. the mode with the highest weekly frequency. Therefore, the merged modes considered were named (1) non-motorized, (2) Private motorized, and (3) Public transport. The modal split data is revealed in Table 2. As in Table 1, for each variable, the sum of the elements sample sizes equals the final quota of the respondents.

**Table 2:** Modal split characteristics and mobility tools ownership.

Variable	Element	Sample	Percentage
Current commuting travel mode before experiment (RP)	Non-motorized	86	15.81
	Private motorized	222	40.81
	Public transport	236	43.38
Intended commuting travel mode after experiment (SP)	Non-motorized	69	12.68
	Private motorized	169	31.07
	Public transport	306	56.25
Cars per household	0	74	13.6
	1	296	54.41
	2	144	26.47
	3 or more	30	5.51
Time travel to work (minutes)	<10	7	1.29
	10–20	40	7.35
	21–30	69	12.68
	>30	428	78.68
Travel distance to work (kilometers)	<5	416	76.47
	5–10	108	19.85
	>10	20	3.68

Information on the place of residence and work was requested, but this data was considered sensitive, and therefore is not shared within the dataset. Nevertheless, the associated spatial features that include distances, consider the location of respondent origin and destination within the study area, correlated with the relative position of downtown area, and the bus station locations. Accordingly, population density was extracted from the geographic information system (GIS) model, as well as the accessibility of public transport stations to respondent's home and workplace and were shared in the dataset. This information was synthesized in Table 3. For each variable, the sum of the elements sample sizes equals the final quota of the respondents.

**Table 3:** Spatial characteristics derived from the GIS model.

Variable	Element	Sample	Percentage
Origin to public transport station	<400 m	511	93.93
	400 … 800 m	31	5.7
	>800 m	2	0.37
Destination to public transport station	<400 m	522	95.96
	400 … 800 m	20	3.68
	>800 m	2	0.37
Origin to downtown area (Euclidian distance, kilometers)	<1	25	4.6
	1–2	109	20.04
	2–3	213	39.15
	3–4	145	26.65
	4–5	15	2.76
	>5	37	6.8
Destination to downtown area (Euclidian distance, kilometers)	<1	41	7.54
	1–2	104	19.12
	2–3	114	20.96
	3–4	104	19.12
	4–5	55	10.11
	>5	126	23.16
Origin - Destination route through downtown area	Yes	167	30.7
	No	377	69.3
Population density at origin (inhabitants per sqkm)	<4000	115	21.14
	4000–6000	58	10.66
	6000–8000	36	6.62
	8000–10000	96	17.65
	10000–12000	48	8.82
	>12000	191	35.11
Population density at origin (inhabitants per sqkm)	<2000	102	18.75
	2000–4000	119	21.88
	4000–6000	80	14.71
	6000–8000	82	15.07
	8000–10000	61	11.21
	>10000	100	18.38

Attitudinal questions were selected from the questionnaire to capture opinions related to the adequacy of the public transport network, car dependency, and issues related to traffic congestion within metropolitan area (see Table 4).

**Table 4:** Attitudinal statements regarding traffic and transport system.

	Fully disagree (%)	Disagree (%)	Agree (%)	Fully Agree (%)	No Response (%)
Inappropriate public transport network	18.01	23.53	34.74	19.12	4.6
Car necessary in daily life	21.14	17.65	18.2	42.1	0.91
Traffic is congested in the city	2.39	2.76	14.34	79.78	0.73

## 2 Experimental design, materials, and methods

We employed a combined estimation method between Revealed Preferences (RP) and Stated Preferences (SP). While RP-data models describe real-life choices and represent actual travel behavior, SP-data-based choice experiments set hypothetical alternatives and record individuals' preferences [5]. The adopted methodology uses disaggregated data on respondents’ travel behavior and related individual data and provides rich behavioral predictions [[6], [7], [8], [9]]. This modelling methodology has been proven to be highly effective in determining the role of selected variables in the selection process and identifying the effects of new policy interventions within transportation sector [10,11].

When modeling RP and SP as random utility models with discrete choices, the utility associated with each transport mode can be expressed as an additive function between regressor vectors describing characteristics $X_{i}$ of an individual *i*, and characteristics $Y_{ij}\text{ and }Y_{ik}$, of the transport alternatives *j* or *k*, and characteristics of the specific effect of the SP experiment ($Z_{ik}$), scaled with respect to corresponding parameter vectors $\alpha_{i}$, $\beta_{ij}$, $\beta_{ik}$ and $\gamma_{ik}$, respectively. The RP and SP models could be jointly estimated and subsequently maximizing the log-likelihood of the following function:

$$L\left(\alpha ,\beta ,\gamma \text{|}X,Y,Z\right)=\prod_{\forall \;i\in I}\prod_{\forall j,k\in J,K}P_{ij}^{RP}{\left(\alpha ,\;\beta \text{|}X,Y\right)}^{\delta_{ji}}\;P_{ik}^{SP}{\left(\alpha ,\beta ,\gamma ,\mu \text{|}X,Y,Z\right)}^{\delta_{ki}}$$

where $\delta_{ji}$ and $\delta_{ki}$ represent modal choice indicators (=1 if alternatives j and k are selected by an individual i in the RP and SP models, respectively; and 0 otherwise), and scale parameter μ. $P_{ij}^{RP}$ and $P_{ik}^{SP}$ represent the marginal probabilities of the selection of the *j* or *k* transport mode, in RP, and SP model respectively. The unknown parameters within the three mode-choice models (RP, SP, and the combined RP–SP) were estimated by using the GAUSS econometric software (version 3.2.32).

The commuting data employed in this paper were gathered from a cross-sectional survey conducted in the CNMA in July 2015 by means of a computer assisted telephone interview (CATI). The SP section was based on the experimental design intended to test the respondent's likelihood to change the current commuting habits over the introduction of the alternative ticketing policy. The alternatives are characterized by a set of relevant attributes and must offer clear and simple choices to the respondent [12,13]. The attributes of the proposed policy included a monthly pass consisting of (1) type, with 2 assigned levels, (2) price, with 3 assigned levels, and (3) incentive bonus, with 3 assigned levels. The public transport types of monthly tickets considered were the two-line and the all-line passes. For each type of monthly pass, 3 levels of pricing were considered, a low, medium, and high costs. The bonus values were considered as percentage of the monthly cost, and set to 2, 5 and 15% points. A full factorial design was employed to generate 18 cases of preference, as a combination of the attributes, as seen in Table 5. Out of the total 18 scenarios for the transport policy alternative, each respondent was presented a single case to accept or reject during the interview.

**Table 5:** Alternative scenarios for public transport.

Case no.	Monthly Cost (RON)	Monthly Pass Type	Bonus (% of cost)
1	55	2 lines	2%
2	55	2 lines	5%
3	55	2 lines	15%
4	70	2 lines	5%
5	70	2 lines	15%
6	70	2 lines	2%
7	90	2 lines	15%
8	90	2 lines	2%
9	90	2 lines	5%
10	85	All line	2%
11	85	All line	5%
12	85	All line	15%
13	110	All line	5%
14	110	All line	15%
15	110	All line	2%
16	135	All line	15%
17	135	All line	2%
18	135	All line	5%

The combined estimation of RP and SP models reveals the values and significance levels of certain coefficients and helps identify the role of certain variables in the choice process [14]. This study assesses differences between generations and their role in tailoring travel demand in emerging urban areas. In this way, several models can be customized by specific socio-economic and demographic features, in order to reveal idiosyncrasies between groups of interest.
