# Supplementary material for: Dataset on commuting patterns and mode-switching behavior under prospective policy scenarios for public transport
Source: Data Brief. 2019 Nov 1;27:104703. doi: 10.1016/j.dib.2019.104703 (PMC6859215; doi:10.1016/j.dib.2019.104703)
Supplement: Multimedia component 3 [file mmc3.docx]

**Survey Instrument**

# <Screening stage>

The purpose of this survey is to obtain information about various aspects of citizen travel behavior for commuting and shopping, and to improve the quality of urban transportation. All data collected from your answers will be processed numerically. Please be assured that any personal information in the responses will not be disclosed under any circumstances.

Q0: Are you over 18 years old, have a part-time job, or a fulltime employee? Are you willing to participate in this research study, to make public transport more attractive and competitive, and to improve urban quality of life? It will take approximately 15 Minutes.

YES NO

# <Commuting trip >

A trip within this survey is defined as the movement performed by an individual, with a specific purpose from an origin to a destination, by different means of transport. It can be initiated by a regular, mandatory action, such as job – as referred as “commuting” – or determined by certain needs, such as shopping or leisure – as referred as “shopping or leisure” within this questionnaire.

Q1: Please state the number of days per week on which you are commuting.

{natural positive number between 0… 7}

We will ask about your current commuting behavior (also for part-time job or students) with several transport modes, from public transport, car/taxi/motorcycle, bicycle and walking.

Q2. Tell us the weekly frequency of commuting trips whose major part is traveled by public transport.

{natural positive number between 0… 7}

Q2-1. State which types of public transport you use. Check among the following.

Bus / Tram / Trolley

Q3. Tell us the weekly frequency of commuting trips whose major part is traveled by car / motorcycle / taxi.

{natural positive number between 0… 7}

Q4. Tell us the weekly frequency of commuting trips whose major part is traveled by bicycle.

{natural positive number between 0… 7}

Q5. Tell us the weekly frequency of commuting trips whose major part is traveled by walking.

{natural positive number between 0… 7}

Q6. Tell us the weekly frequency of commuting trips whose major part is traveled by other means.

{natural positive number between 0… 7}

Q6-1. Tell us the mean of transport used.

{verbatim}

The Cluj-Napoca Company of public transport (CTP) is facing modernization of its infrastructure and planning to introduce personalized contactless cards, which can be charged with individual trips, weekend and monthly passes. Therefore, this research objective is to estimate user’s perceptions on the presumed ticket types and fares. *Any numbers or factual conditions in the questions are based on current realities and are hypothetical. They are not meant to be part of an actual plan of action.* For the transport card charges, we assume the award of compensations, as percentage of the ticket price. The bonus points can be accumulated and used for tickets and monthly passes for PT, as well as payment method for different services. The more you travel by public transport, the more points you earn and you can use them to enjoy public transport company’s and merchants’ services.

## SP survey 1 (weekday commuting)

Please think of the following type of monthly pass. Listen to the characteristics and please tell us if you would use such pass, considering your travel needs. Listen carefully to the **type** (scope) **of pass**, the **price** and corresponding awarded **bonus** **points**, expressed in percent of the price.

***{ONE case is RANDOMLY revealed to the responded}***

Q7: Would you buy such ticket?

1.Yes　２. No

Q8. Will you increase the frequency of commuting trips by public transport?

YES / NO

Q8-1. Tell us the weekly frequency of commuting trips by public transport.

{natural positive number between 0… 7}

Q9. Will you decrease the frequency of commuting trips by other transportation means?

YES / NO

Q9-1. Which transportation means will be decreased and specify the decreased frequency.

| Mode | Car/Motorcycle/ Taxi | Bicycle | Walk | Other |
| --- | --- | --- | --- | --- |
| Decreased frequency |  |  |  |  |

# <Public transport satisfaction>

**Q10. Please answer the user level of information available that makes you aware of the public transport service characteristics in Cluj-Napoca.** *Answer from a scale to 1 (total agreement) to 4 (total disagreement).*

I have information about the structure of the transit route map (station location, routes).

I have information about the operating hours of public transport vehicles.

I have information about the frequency of the public transport vehicles.

I have information about the transfers needed to get to a destination.

**Q11: Please answer to the general questions on public transport.** *Answer from a scale to 1 (total agreement) to 4 (total disagreement).*

I think that public transportation became a loss-making operation and bus routes are out of date because more people travel by cars.

I think the public transport such as bus, trolley or tram is absolutely necessary in daily life.

I think that public transport fares are cheap.

I think that the walking time from home to the relevant station for my commuting by PT is short.

Waiting time spent in stations for the PT vehicles is long.

I think public transport vehicles at the time of commuting are crowded.

I think bus stations layout (shelter, seats, lights) is satisfactory.

# <Traffic and environment perceptions>

**Q12. We will ask about road traffic situation in your city.** *Answer from a scale to 1 (total agreement) to 4 (total disagreement).*

The car is absolutely necessary in daily life for me.

I believe traffic is congested in my city.

I think that it is dangerous and hard to walk because there are too many cars.

I think the current local government transport policy is correct.

**Q13: Please answer the following questions on environmental awareness and behavior.** *Answer from a scale to 1 (total agreement) to 4 (total disagreement).*

I always take environmental friendly actions (eco driving, garbage disposal, electricity use, etc.).

I think that the car is a major cause of global warming.

I like driving.

I would use Bike-sharing system in Cluj for commuting.

I would use Bike-sharing system in Cluj for shopping.

I would use Bike-sharing system in Cluj for leisure.

# < Socio-economic data>

**The following questions will be essential in our analysis. Please tell us about you.**

Q14: Your home location (name of street, number interval or close landmark)

Q15：Gender

１．Male　　　　　２．Female

Q16：Age

Q17：Final educational background

１． Elementary School ２． Junior High School　　３． Senior High School ４． Bachelor

５． Master　　 ６． Ph.D ７． Others (…….)

Q18：Household monthly income (net)

１． Below 1000 RON ２． 1001 – 1500 RON ３． 1501 – 2000 RON

４． 2001 – 2500 RON ５． 2501 – 3000 RON ６． 3001 – 3500 RON

７． 3501 – 4000 RON ８． 4001 – 4500 RON ９． Above 4500 RON

Q19：Your household monthly transportation expenditure.

１． Below 50 RON ２． 51 – 100 RON ３． 101 – 150 RON

４． 151 – 200 RON ５． 201 – 250 RON ６． 251 – 300 RON

７． 301 – 350 RON ８． 351 – 400 RON ９． Above 400 RON

Q20: Occupation

１． Government employee　 　 2． Non. Government Employee 　3. Not answering

Q20a. Occupational Field

Finance; Sales, food industry; Transport; Real estate; Services; Industry; IT; Not answering

Q21: Your work location (name of street, number interval or close landmark)

Q22: Do you have a driver license?

１．Yes　　　　　２．No

Q23：Number of cars for your household

1. 0　 　２． 1 3． 2 4． 3 or more

Q24: Household size (including yourself)

1.　1　 　２． 2 3． 3 4． 4 or more

Q25: Marital status:

1.　Married　 　２． Not married

Q26: Type of dwelling:

1. Apartment 　２． Individual house
