# Supplementary material for: Dataset on commuting patterns and mode-switching behavior under prospective policy scenarios for public transport
Source: Data Brief. 2019 Nov 1;27:104703. doi: 10.1016/j.dib.2019.104703 (PMC6859215; doi:10.1016/j.dib.2019.104703)
Supplement: Multimedia component 4 [file mmc4.doc]

@RP/SP Model@

new;

@Reading data set@

load x[546,54] =C:\...\samp_red.csv;

@For removing the header of the file@

x=x[3:546,.];

@x=delif(x,x[.,2].==1);@ @This sentence is for delete the data with 1 in 7th column (it is not used in this code)@

@x=delif(x,x[.,2].==2);@

@x=delif(x,x[.,2].==3);@

@x=delif(x,x[.,2].==4);@

@x=delif(x,x[.,2].==5);@

@x=delif(x,x[.,2].==6);@

@x=delif(x,x[.,2].==7);@

@Calling library of maximum likelihood@

library maxlik;

#include maxlik.ext;

maxset;

@setting output@

output file=C:...\RPSP01al.out on; @<-- change address@

__output=2; @This sentence is for not overwriting a new result on the last result@

@Setting maximization algorithm@

@_max_MaxIters=700;@

_max_Algorithm= 2;

@4=NR 5=BHHH@

@defolt:_max_GradTol = 1e-5;@

@_max_GradTol = 1e-6;@ @This value should not be larger! To be smaller is not problem.@

_max_CovPar=0;

@defining argument of the function@

clearg Vwr,Vcr,Vbr,Vws,Vcs,Vbs; @Utility functions r=RP, s=SP@

clearg Ewr,Ecr,Ebr,Ews,Ecs,Ebs;

clearg sel_Er,sel_Es,probr,probs,prob,para;

@Defining parameters@

@The number of characters in the name of parameter should be up to 8 characters.@

let _max_parnames=

@choice specific variables@

"cons_wak" @1 constant for walk mode RP@

"cons_car" @2 constant for bus mode RP@

"tra_time" @3 travel time, in minutes@

@individual specific variables@

"age>45na" @4 age > 45 yrs walk & bus@

"carperhh" @5 nr of cars per household > 1@

"gender_c" @ 6 gender@

"income_b" @7 hh income, bus@

"occup_nm" @8 occupation walk and bus@

"lice_bus" @9 licence bus@

"income_c" @10 hh income, car@

@spatial characterstics@

"discbdwk" @11 distance Origin - destination - walk@

"thrcbd_b" @12 through CBD 1km buff - bus@

@SP PT parameters@

"prc_tk_b" @13 price ticket @

@scale parameter@

"myu_scal" @14 scale parameter@

"k_bus_sp" @15 cost car@

"orig2PTs" @16 origin to PT stop@

"marrid_b" @17 marital status bus@

"typ_tikb" @18 type tiket@

"badtrntw" @19 bad pt network 1 - dissagree 4 - total agree@

"auto_nec" @20 auto necessary 1 - dissagree 4 - total agree@

"Tr_congt" @21 traf cong in the city 1 - dissagree 4 - total agree@

"bonus_tk" @22 bonus ticket@

;

@start of maximum likelihood procedure@

proc li(b,x);

@Set of utility functions@

@utility for non motorized modes REVEALED PREFERENCES@

Vwr= b[1] @constant walk@

+b[3]*(x[.,47]) @travel time in minutes@

+b[8]*x[.,14] @occupation@

+b[4]*(x[.,10].>=48) @age above 48 yrs@

+b[11]*(x[.,33].<1000) @ O-D < 1 km @

+b[21]*(x[.,54].>=4) @ dangerous to walk in the city @

;

@utility for car mode@

Vcr= b[2] @constant bus@

+b[3]*(x[.,45]) @travel time in minutes@

+b[5]*(x[.,16].>1) @number cars@

+b[10]*ln(x[.,12]./x[.,17]/1000) @Household income@

+b[6]*(x[.,9]) @ male@

+b[16]*ln(x[.,29]) @distance Origin to PT@

+b[20]*(x[.,49].==4) @ auto is neccesary in daily life@

;

@utility for PT mode@

Vbr= b[3]*(x[.,46]) @travel time in minutes@

+b[8]*x[.,14] @occupation@

+b[9]*x[.,15] @driver licence@

+b[7]*ln(x[.,12]./x[.,17]/1000) @Household income per individual@

+b[4]*(x[.,10].>=48) @age above 48 yrs@

+b[12]*x[.,34] @O-D line pass through CBD@

+b[17]*(x[.,18]) @marital status, bus@

+b[19]*(x[.,53].>=3) @bad organized PT network@

;

@utility for non motorized modes STATED PREFERENCES@

Vws= b[14]*(b[1] @constant walk@

+b[3]*(x[.,47]) @travel time in minutes@

+b[8]*x[.,14] @occupation@

+b[4]*(x[.,10].>=48) @age above 48 yrs@

+b[11]*(x[.,33].<1000) @ O-D < 1 km @

+b[21]*(x[.,54].>=4) @ dangerous to walk in the city @

)

;

@utility for car mode@

Vcs= b[14]*(b[2] @constant bus@

+b[3]*(x[.,45]) @travel time in minutes@

+b[5]*(x[.,16].>1) @number cars@

+b[10]*ln(x[.,12]./x[.,17]/1000) @individual Household income@

+b[6]*(x[.,9]) @ male@

+b[16]*ln(x[.,29]) @distance Origin to PT@

+b[20]*(x[.,49].==4) @ auto is neccesary in daily life@

);

@utility for PT mode@

Vbs= b[14]*(

b[15] @SP specific constant@

+b[3]*(x[.,46]) @travel time in minutes@

+b[8]*x[.,14] @occupation@

+b[9]*x[.,15] @driver licence@

+b[7]*ln(x[.,12]./x[.,17]/1000) @Household income per individual@

+b[4]*(x[.,10].>=48) @age above 48 yrs@

+b[12]*x[.,34] @O-D line pass through CBD@

+b[17]*(x[.,18]) @marital status@

+b[19]*(x[.,53].>=3) @well organized PT network@

+b[13]*ln(x[.,7]) @price ticket@

+b[18]*(x[.,6]) @type ticket 2 lines@

+b[22]*ln(1+x[.,8]/x[.,7]) @bonus ticket @

)

;

Ewr=x[.,20].*exp(Vwr);

Ecr=x[.,22].*exp(Vcr);

Ebr=x[.,21].*exp(Vbr);

sel_Er=Ewr.*(x[.,3].==3)+Ecr.*(x[.,3].==1)+Ebr.*(x[.,3].==2); @selection of RP chosen modes@

Ews=x[.,20].*exp(Vws);

Ecs=x[.,22].*exp(Vcs);

Ebs=x[.,21].*exp(Vbs);

sel_Es=Ews.*(x[.,4].==3)+Ecs.*(x[.,4].==1)+Ebs.*(x[.,4].==2); @selection of SP chosen modes@

probr=sel_Er./(Ewr+Ecr+Ebr);

probs=sel_Es./(Ews+Ecs+Ebs);

prob=probr.*probs;

retp(ln(prob));

para=rows(b);

endp;

@end of maximum likelihood procedure@

@innitial value of parameters@

start=zeros(22,1);

@

start={

-3.615844

-5.291775

-0.72934

0.399666

1.001201

1.055653

-0.423153

-0.821288

-1.549662

0.340578

1.032675

0.545647

-1.528831

0.768509

7.486601

0.319996

-0.480522

0.489907

0.529837

1.566863

-0.433344

0.601279

}

;@

@maximum likelihood procedure@

{b,ff,gg,cov,retcode}=maxlik(x,0,&li,start);

call maxprt(b,ff,gg,cov,retcode);

@Sample enumeration@

"Sample enumeration RP";

"Walk"; meanc(Ewr./(Ewr+Ecr+Ebr)*100);

"Car"; meanc(Ecr./(Ewr+Ecr+Ebr)*100);

"Bus"; meanc(Ebr./(Ewr+Ecr+Ebr)*100);

@Sample enumeration SP@

"Sample enumeration SP";

"Walk"; meanc(Ews./(Ews+Ecs+Ebs)*100);

"Car"; meanc(Ecs./(Ews+Ecs+Ebs)*100);

"Bus"; meanc(Ebs./(Ews+Ecs+Ebs)*100);

@summary of statistics@

ff;

format /m1 /rdn 7,5;

"Initial log Likelihood"; @Initial LL@

ff1=ln(1/3)*rows(x)*2;

ff1;

"Log likelihood at convergence";ff*rows(x); @LL at convergence@

"Rho-squared";(ff1-ff*rows(x))./ff1;

"Adjusted Rho-squared"; @Adjusted Rho Sq@

ad_rho=(ff1-(ff*rows(x)-rows(b)))./ff1;

ad_rho;

"AIC"; @AIC@

-2*ff*rows(x)+2*rows(b);

"BIC"; @BIC@

-2*ff*rows(x)+(rows(b)*ln(rows(x)));

output off;

end;
